# Neurotrophic factor alpha 1 gene therapy in Alzheimer’s disease: scope and advancements

**DOI:** 10.3389/fnmol.2025.1518868

**Published:** 2025-04-01

**Authors:** Ammara Shaikh, Fairus Ahmad, Seong Lin Teoh, Jaya Kumar, Mohamad Fairuz Yahaya

**Affiliations:** ^1^Department of Anatomy, Faculty of Medicine, Universiti Kebangsaan Malaysia, Kuala Lumpur, Malaysia; ^2^Department of Physiology, Faculty of Medicine, Universiti Kebangsaan Malaysia, Kuala Lumpur, Malaysia

**Keywords:** Alzheimer’s disease, amyloid *β* peptide, neurotransmitter system (NTS) abnormalities, neurotrophic factor alpha 1 (NF-α1), carboxypeptidase E, gene therapy in AD

## Abstract

Alzheimer’s disease (AD) is the leading cause of dementia, accounting for 60–80% of all cases globally. Hallmark pathologies of AD include the accumulation of amyloid *β* peptide and phosphorylated tau, leading to neuronal circuit dysfunction, defective axonal transport, and neurotransmitter system (NTS) abnormalities. Disruptions in acetylcholine, GABA, dopamine, serotonin, and glutamate levels, as well as the loss of cholinergic, GABAergic, and monoaminergic neurons, contribute to the progression of AD. Additionally, neurotrophic factors like brain-derived neurotrophic factor (BDNF) and nerve growth factor (NGF) are significantly reduced in AD, impacting neuronal health and synaptic integrity. This review highlights the emerging role of neurotrophic factor alpha 1 (NF-α1), also known as carboxypeptidase E, in AD. NF-α1 shows neuroprotective and neurogenesis-promoting properties, offering potential for therapeutic interventions. The review compares NF-α1 gene therapy with other neurotrophin-based treatments, providing insights into its efficacy in AD management.

## 1 Introduction

Alzheimer’s disease (AD) is the most common cause of dementia accounting for 60–80% of all cases worldwide (cdc.org, 2022). The disease is characterized by the extraneuronal accumulation of amyloid *β* peptide (Aβ) and an intraneuronal deposition of phosphorylated tau (p-tau) (Wang et al., 2024; Arrozi et al., 2022). The neuropathology of AD also involves dysfunction of the neuronal circuits. These abnormalities result from defective axonal transport, aberrant synaptic morphology, and neurotransmitter systems (NTS) dysfunction. The insults to the NTS comprise low levels of acetylcholine (ACh), gamma-aminobutyric acid (GABA), and monoamines (dopamine and serotonin) along with increased levels of glutamate. Moreover, there is a loss of cholinergic, GABAergic, and monoaminergic neurons. The defects in synaptic morphology and NTS are a consequence of the tau and amyloid pathologies (Decker et al., 2015; Palop et al., 2007; Shaikh et al., 2023a; Yang et al., 2023). Besides, the defective neuronal transport is either due to disruption of cytoskeleton or disturbance of neurotrophic factors; the damage to neuronal cytoskeleton results due to destabilization and aggregation of cytoskeleton proteins secondary to neurofibrillary tangle (NFT) formation. The disrupted cytoskeleton compromises axonal transport, and can subsequently lead to neurodegeneration.

In addition, there is a relatively overlooked element of AD pathology, which is damage to neurotrophins. The neurotrophin family is composed of several neurotrophic factors, most prominent are brain-derived neurotrophic factor (BDNF), nerve growth factor (NGF), neurotrophin-3 (NT-3) and neurotrophin-4/5 (NT-4/5) (Omar et al., 2022; Skaper, 2018). Out of the mentioned factors, BDNF and NGF are found to be markedly reduced in AD brain, hence, playing an important role in AD pathogenesis. Generally, the neurotrophic factors are responsible for a number of physiological actions including maintaining neural cell proliferation, homeostasis and function. Therefore, their abnormality result in neuronal dysfunction, synaptic aberration and abnormalities of neuronal circuits. A number of studies are conducted in last decade that deduced abnormality of neurotrophic factors in AD. Moreover, a newly identified member, known as neurotrophic factor alpha 1 (NF-α1) or carboxypeptidase E has recently garnered attention due to its neuroprotective (Cheng et al., 2013), neurogenesis-promoting (Cheng et al., 2015), and dementia, AD and depression attenuating potential (Cheng et al., 2015, 2016; Xiao et al., 2023). This review will, therefore, focus on the advancement in understanding the role of NF-α1 and comparison of NF-α1 gene therapy with other neurotrophin-targeted therapies for the treatment of AD.

## 2 Physiological functions of neurotrophins

Among the neurotrophic factors that regulate the development, survival, and overall health of the nervous system, neurotrophins is the name assigned to four closely related factors that have well-established roles in maintaining normal neural functions. These four neurotrophins are NGF, BDNF, NT-3, NT-4/5. Neurotrophins modulate several activities in the central nervous system (CNS) and peripheral nervous system (PNS). That said, NGF exerts its effects on cell count of various types of cells within and outside the CNS. NGF is abundantly expressed by neurons, oligodendrocytes, microglia, and astrocytes. It is specifically crucial for the maintenance, survival, and function of the cholinergic neurons in the basal forebrain (BFCN). Additionally, it also influences PNS by modulating neuronal count and activity of the sympathetic and sensory neurons (Niewiadomska et al., 2011). Likewise, BDNF is abundant in both CNS and PNS with merely detectible levels in heart, lung and skeletal muscles (Miranda et al., 2019). BDNF functioning is essential in the developing as well as in adult CNS. In the developing CNS, BDNF is crucial for the survival and differentiation of neuronal populations, whereas, later in the adult CNS, it regulates synaptic plasticity and transmission (Bathina and Das, 2015). On the other hand, BDNF in the PNS is associated with growth and survival of peripheral sensory neurons, as well as post-injury defense, such as neuronal survival, axonal growth and regeneration (Miranda et al., 2019).

Although less studied compared to other neurotrophins, NT-3 and NT-4/5 promote the survival and differentiation of existing neurons, and the growth and differentiation of new neurons. NT-3 is widely expressed in the hippocampus and facilitates hippocampal plasticity and neurotransmission by regulating neurogenesis (Shimazu et al., 2006). The neurotrophins exert their effects by binding to the tropomyosin-related kinase (Trk) receptors. NGF binds to the TrkA receptor, NT-3 bind to TrkC receptors, whereas both BDNF and NT-4/5 show high binding affinity for TrkB recptors. Besides, all Trk receptor-binding neurotrophins (Numakawa and Kajihara, 2023), especially NGF, have low affinity for the neurotrophin receptor p75 (p75NTR). It is suggested that NGF has the highest binding affinity for p75NTR in comparison to other neurotrophins. Regardless of the type, ligand binding to the p75NTR results in neuronal apoptosis (Sajanti et al., 2020).

The newly identified member NF-α1 is present in high concentration in the human brain, especially in hypothalamus, paraventricular nucleus, pituitary, hippocampus, and amygdala (Xiao et al., 2019). In hippocampus, NF-α1 promotes hippocampal neurogenesis (Xiao et al., 2019) and controls TrkB surface delivery in hippocampal neurons. By acting as a regulated secretory pathway sorting receptor, NF-α1 modulates long-term potentiation stimuli-induced TrkB surface insertion and plays an important role in TrkB-dependent synaptic plasticity and hippocampal-dependent memory modulation (Li et al., 2021).

## 3 Neuronal physiopathology in Alzheimer’s disease—effect on neurotrophins

The commencement of AD is marked by the formation of Aβ and p-tau which soon begin to aggregate as Aβ plaques and NFTs, respectively. Physiologically, tau is a microtubule-associated phosphoprotein that is abundant in neurons, especially in axonal processes. In normal conditions, the attachment of tau to microtubules is essential for the assembly, stabilization, and proper functioning of the latter. By doing so, tau maintains neuronal morphology, regulates axonal transport, and promote neurite outgrowth. Conversely, when the tau is phosphorylated and, therefore, not able to attach to the microtubule, as happens in hyperphosphorylation in AD, there is decreased bundling and stability of microtubules resulting in disturbed neuronal architecture and impaired axonal transport; all of which ultimately lead to cell death (Amadoro et al., 2011; Youdim et al., 2003). On the other hand, Aβ is formed by the cleavage of amyloid precursor protein (APP) by the amyloidogenic pathway. Besides promoting oxidative stress and neuroinflammation, Aβ can also exert neurotoxic effects by disrupting neuronal cytoskeleton (Jarero-Basulto et al., 2024; Lee et al., 2019; Wang et al., 2018).

The Aβ and tau pathologies initiate a vicious cycle of oxidative stress and neuroinflammation, which in turn cause more amyloid and tau deposition, subsequently leading to neuronal insults and neurodegeneration. The neurotoxic effects, along with the cytoskeletal damage (Dong et al., 2022; Wang et al., 2018), are responsible for neuronal loss, including that associated with the NTS (Stratmann et al., 2016). Moreover, there is a reduction of neurotrophins, predominantly BDNF, in brain primarily due to neurodegeneration. As hyperphosphorylated tau decreases BDNF levels (Rosa et al., 2016), which in turn increase Aβ plaque accumulation, NFT deposition (Ginsberg et al., 2019) and neuroinflammation (Wang et al., 2019) may facilitate disease progression by lowering BDNF levels.

NGF, as the name suggests, plays a role in neuronal proliferation, activation, differentiation and survival, especially of BFCN (Aloe et al., 2015). NGF signaling also controls the amyloidogenic pathway and Aβ formation in hippocampal neurons, hence, decrease in NGF results in an overproduction and subsequent aggregation of Aβ plaques. Therefore, lower NGF levels in AD result in neurotoxicity that can lead to degeneration of the BFCN and hippocampal neurons (Matrone et al., 2008). The findings of this newer study are in contrast to the previous report suggesting increase NGF in hippocampus and frontal cortex of post mortem AD brain (Hock et al., 2000). In comparison to BDNF and NGF, the role of NT-3 and NT-4/5 in AD is not yet established. However, a study reported lower levels of NT-4/5 with no change in the NT-3 levels in the hippocampus and cerebellum of the post-mortem AD brain (Hock et al., 2000).

**Figure 1:**
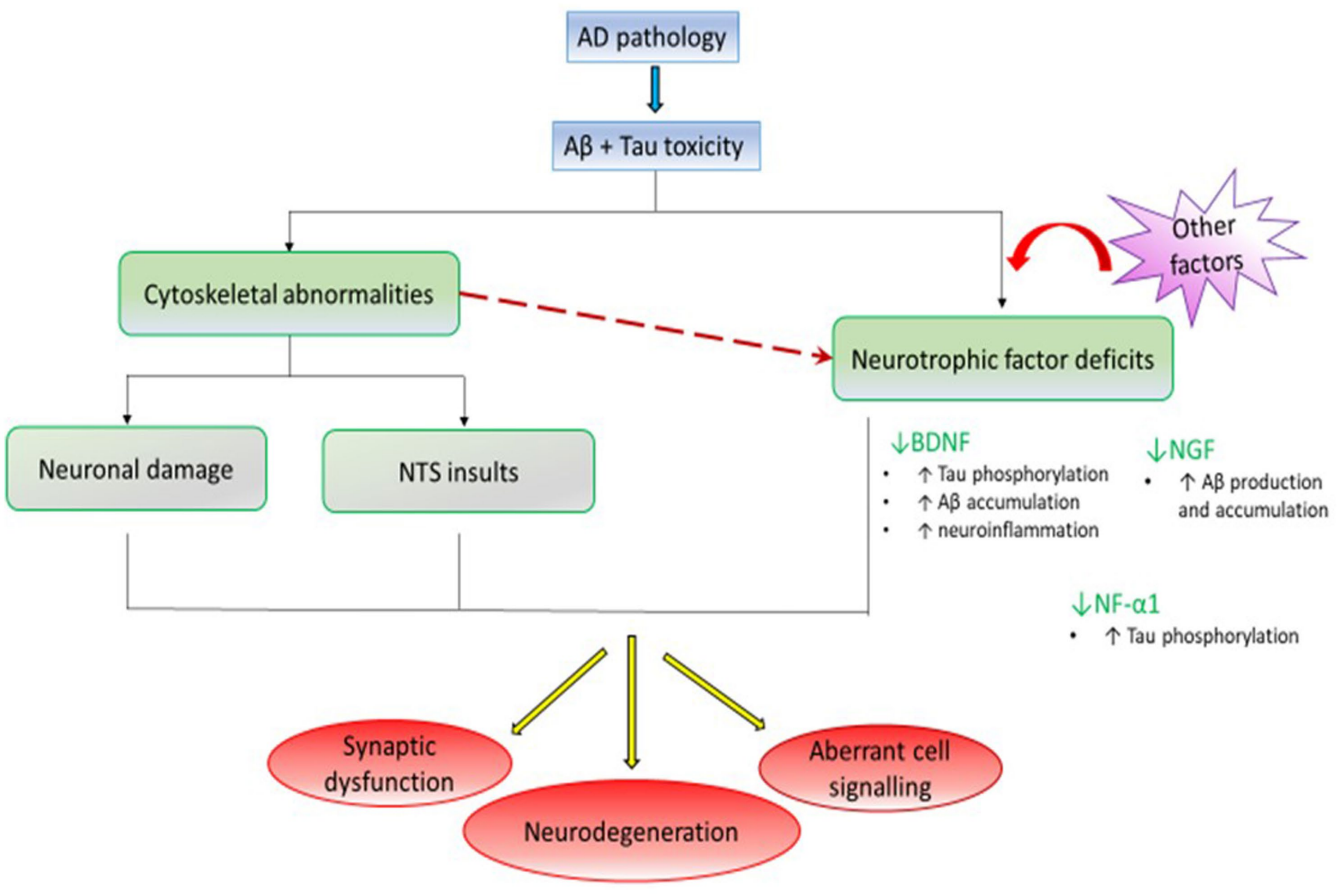
The AD pathology involves Aβ and p-tau accumulation. The p-tau formation along with Aβ deposition disrupts microtubules and causes abnormalities of the neuronal cytoskeleton; the latter of which results in neuronal damage and defective NTS. Moreover, the Aβ and tau toxicity, damaged cytoskeleton of neurotrophic factor-releasing neurons together with other lesser known factors lower the level of neurotrophic factors. This way, the cytoskeletal abnormalities, and neurotrophic factor deficits result in synaptic dysfunction, aberrant cell signaling, and neurodegeneration. Furthermore, decreased concentration of BDNF, NGF, and NF-α1 further worsens AD pathology by promoting neuroinflammation, tau hyperphosphorylation, and Aβ accumulation.

A new study identified a mutation in the NF-α1 gene in AD human brain, which resulted in decreased levels of enzymatically active NF-α1 (Cheng et al., 2016). When the similar abnormal gene expression was reproduced in an NF-α1 gene knock-out mice, the latter showed severely decreased dendrites in the hippocampal cornu ammonis (CA) area 3 (Cheng et al., 2015, 2016) and medial prefrontal cortex, and diminished neurogenesis in the dentate gyrus (DG), along with increased tau hyperphosphorylation at middle-aged stage (50-week age). Moreover, these pathologies were found to be associated with poor memory performance and depressive-like behavior (Cheng et al., 2016) (Figure 1).

## 4 Neurotrophic factor alpha 1

NF-*α*1 is a 476-amino acid multifunctional polypeptide that performs a variety of enzymatic and non-enzymatic functions in endocrine system and CNS. The enzymatic activity involves cleavage of basic residues from carboxyl (C) terminal to generate mature peptide hormones and neuropeptides. Whereas, the non-enzymatic effects include multiple physiological roles in modulating and maintaining normal neural functions in the CNS.

NF-α1 is synthesized as a pre-proNF-α1 consisting of a signal peptide, a catalytic domain, and a C-terminal domain. The pre-proNF-α1 then undergoes cleavage at multiple sites to produce the mature NF-α1 that is comprised of a prohormone sorting signal-binding site, an amphipathic α-helical transmembrane domain, a cytoplasmic tail that interacts with microtubule proteins for vesicle transport and a zinc-binding site in the enzymatic domain. The processing of pre-proNF-α1 begins when the 25-amino acid-long signal peptide directs the proNF-α1 to the cisternae of the rough endoplasmic reticulum, where the former is removed. Next, the proNF-α1is channeled through the Golgi complex to the granules of the regulated secretory pathway, removing the 17-amino acid-long “pro” segment and resulting in the formation of the mature membrane-associated protein (NF-α1 43-476). In the membrane-bound form, the embedding of the C-terminal of NF-α1 in the lipid raft is responsible for its attachment to the trans-Golgi network. This penetration of C-terminal also creates a cytoplasmic tail that interacts with dynactin, dynein and other cytoskeletal proteins to mediate transportation of the granules of the regulated secretory pathway to the plasma membrane, which are then secreted via exocytosis. However, some of the NF-α1 within the granules is further cleaved at Arg455-Lys456 to generate mature soluble NF-α1, which is enzymatically more active than the membrane-bound form (Cawley et al., 2012).

The soluble NF-α1 works as an enzyme that acts on the neuropeptide precursors or prohormones to convert them into their active forms. Whereas, the membrane-bound NF-α1 functions as the sorting receptor that directs the prohormones to the regulated secretory pathway. This way, NF-α1 is responsible for sorting, vesicle transport, and secretion of prohormones, including proBDNF. Moreover, NF-α1 facilitates neuronal activity-dependent TrkB transport from dendritic shafts to the plasma membrane and thereby augments BDNF signaling (Li et al., 2021). Similarly, NF-α1 also modulates NGF-induced neurite outgrowth and, therefore, possibly has an important role in CNS neurodevelopment (Selvaraj et al., 2015). NF-α1 is highly expressed in pyramidal cell layers of CA and granular cells of the DG of the hippocampus, and its expression declines with age (Jiang et al., 2024). Due to its presence, besides playing a role as a prohormone-processing enzyme, NF-α1 is integral in hippocampal neurogenesis and in modulating the mature BDNF/TrkB signaling pathway (Jiang et al., 2024).

## 5 Gene therapy approaches targeting NF-α1: delivery methods and implications

As no neurotrophin can cross the blood–brain barrier (BBB) (Tambaro et al., 2023), the delivery to the brain can be broadly divided into three methods - stereotactic delivery, minimally invasive trans-nasal route, and utilization of focused ultrasound to transiently change BBB permeability to help peripherally injected exogenous neurotrophin reach the brain (Nagahara et al., 2018; Padmakumar et al., 2021; Rafii et al., 2014; Xhima et al., 2022).

Stereotactic injections have been established as a pivotal method for delivering therapeutic agents directly to targeted brain regions, particularly in the context of localized pathologies such as AD. This technique employs a three-dimensional coordinate system that allows for precise targeting, which is crucial for maximizing the local concentration of therapeutic agents, thereby enhancing their efficacy. For instance, a clinical trial demonstrated the safety and feasibility of stereotactic injections of human umbilical cord blood mesenchymal stem cells into the hippocampus of AD patients, highlighting the method’s potential in addressing neurodegenerative conditions (Kim et al., 2015). Furthermore, the ability to achieve high local concentrations of therapeutic agents is underscored by the findings of other studies that emphasize the importance of targeted delivery in improving treatment outcomes for conditions like AD (Tekin, 2024).

However, the invasiveness of stereotactic injections poses significant risks, including infection, hemorrhage, and potential damage to surrounding brain tissues. These complications necessitate specialized surgical expertise, which can limit access to this treatment for many patients (Kim et al., 2015). For example, while stereotactic methods are effective, the associated risks and the requirement for skilled personnel can create barriers to widespread application, particularly in less accessible medical settings (Tekin, 2024). Additionally, while complications can be managed with advanced imaging techniques, the inherent risks of invasive procedures remain a critical consideration in their application for AD models (Malone et al., 2015). Thus, while stereotactic injections represent a promising avenue for targeted therapies in Alzheimer’s, careful consideration of their risks and the need for specialized training is essential for optimizing patient outcomes.

A recent study deduced that the NF-α1 gene delivery in the early stages of AD can halt disease progression. In this study, the NF-α1 gene delivery to the 3xTg-AD mice hippocampus through Adeno-associated viral vector was associated with decreased APP expression and reduced tau hyperphosphorylation (Xiao et al., 2023). The APP levels were observed to be as low as of the non-AD brains which subsequently resulted in reduction of the intracerebral burden of insoluble Aβ_1-42_. The cumulative outcome of the NF-α1 gene therapy was its potential ability to reduce the pathologic burden of disease and exert neuroprotective effects on the pyramidal neurons, which was evident in the behavior as improved cognition of the transgenic mice (Xiao et al., 2023).

Another innovative delivery method is intranasal delivery, which is recognized for its ease of administration and potential to enhance patient compliance. This method allows for rapid access to the CNS by bypassing the BBB, which is a significant advantage in treating neurological conditions (Kulkarni et al., 2023). However, this delivery method has limitations, including the restricted volume of drug that can be effectively administered and variability in absorption rates, which can lead to inconsistencies in achieving therapeutic concentrations (Madden et al., 2023).

A newer study has utilized both intracerebral and intranasal routes to deliver agomirs that upregulate NF-α1 expression (Jiang et al., 2024). Agomirs are chemically altered double-stranded microRNA (miRNA) mimics designed to replicate the function of mature endogenous miRNAs after being introduced into cells. By engaging the cell’s natural miRNA machinery, they enhance the activity of endogenous miRNAs (Jiang et al., 2024). In their study, Jiang et al. (2024) deduced that the intranasal instillation of NF-α1 agomirs is as effective as intracerebroventricular (icv) injection to upregulate NF-α1 expression in the APP/PS1 hippocampus. Restoring NF-α1 was associated with improved BDNF maturation and increased hippocampal neurogenesis, which attenuated cognitive deficits in the APP/PS1 transgenic mice. Both induction methods augmented synaptic integrity by increasing synaptophysin, and promoting dendritic lengthening and complexity. Moreover, they resulted in decrease burden of amyloid and tau pathologies in hippocampus of the transgenic mice. By these mentioned mechanisms, the intranasal and icv administration of agomirs improved cognitive impairments in the APP/PS1 mice (Jiang et al., 2024).

In recent years, non-invasive techniques such as focused ultrasound (FUS) have gained traction as alternative delivery methods. FUS utilizes high-frequency sound waves to temporarily disrupt the BBB, enabling therapeutic agents to penetrate the CNS more effectively (Gorick et al., 2022). This approach is attractive because it minimizes the risks associated with invasive procedures. Moreover, the real-time imaging capabilities of FUS allow for precise targeting and monitoring during the delivery process (Meng et al., 2021). However, variability in patient anatomy and the specific parameters used can affect its overall effectiveness, highlighting the need for tailored approaches to maximize outcomes.

## 6 Mechanistic insights of NF-α1 action

NF-α1 exerts significant neuroprotective effects through various mechanisms that are critical for addressing the downstream consequences of AD pathology. One of the primary actions of NF-α1 involves the modulation of BDNF and its receptor, TrkB, which are essential for promoting synaptic plasticity and survival of neurons. NF-α1 enhances BDNF/TrkB signaling, leading to increased synaptic strength and resilience against neurodegenerative processes (Liu et al., 2023). Additionally, NF-α1 has been shown to suppress the activation of microglia and astrocytes, which are often hyperactivated in the AD brain and contribute to neuroinflammation and neuronal damage (Shaikh et al., 2023b; Wu and Eisel, 2023). By attenuating the inflammatory response mediated by these glial cells, NF-α1 can mitigate the adverse effects of neuroinflammation, thereby protecting neuronal integrity (Xiao et al., 2023; Jiang et al., 2024). Furthermore, NF-α1 plays a role in regulating apoptotic pathways, particularly through the modulation of Bcl-2 and Bax proteins. By promoting the expression of anti-apoptotic factors such as Bcl-2 and inhibiting pro-apoptotic factors like Bax, NF-α1 can reduce neuronal apoptosis, which is a hallmark of AD progression (Xiao et al., 2023). Collectively, these mechanisms underscore NF-α1’s potential to counteract synaptic loss and neuronal death, two critical features of AD. Furthermore, NF-α1 modulates dopaminergic system by increasing trafficking of dopamine transporter to the presynaptic membrane of dopaminergic neurons (Zhang et al., 2009). Therefore, the NF-α1 gene therapy may enhance dopamine uptake and availability, and improve dopamine related aspects of the disease pathogenesis (Shaikh et al., 2023a). By enhancing synaptic plasticity, reducing neuroinflammation, and inhibiting apoptosis, NF-α1 could effectively address the multifactorial aspects of AD pathology, thereby offering a promising therapeutic strategy for this debilitating disease.

## 7 Comparison with other neurotrophic factors gene therapies

### 7.1 Brain-derived neurotrophic factor (BDNF)

BDNF is the most studied neurotrophic factor. The BDNF gene delivery in the entorhinal cortex is found to increase BDNF levels (Nagahara et al., 2009, 2018) and decrease Aβ plaque accumulation (Tambaro et al., 2023). Besides, it is also found to reverse APP-related aberrant gene expression, while improving synaptic dysfunction, cell signaling and preventing neuronal loss (Nagahara et al., 2009). Thus, BDNF administration compensates for genetic, physiological and behavioural aspects of AD-related neuronal damage. Moreover, as BDNF produced in the entorhinal cortex also reaches the hippocampus via anterograde transport (Nagahara et al., 2018), the neurotrophin can also attenuate neuronal damage in the latter. These effects collectively appear as restoration of learning and memory (Nagahara et al., 2009). Taken together, the studies on BDNF delivery to the entorhinal cortex provide a convincing rationale of ameliorating entorhinal and hippocampal damage, thereby, treating AD symptoms and neuropathology. Although useful, the brain delivery of BDNF is associated with adverse effects, such as neuropathic pain and seizures (Li et al., 2023). To address this issue, another approach utilized the BDNF messenger RNA (mRNA)-based therapy, in which the engineered mRNA was delivered into the brain and translated into astrocytes, which minimized the occurrence of seizures (Li et al., 2023).

The study comparing NF-α1 and BDNF showed that the former is better in neuroprotection and improving cognition compared to the latter. The study deduced that NF-α1 is more critical than BDNF in protecting CA3 pyramidal neurons against stress-induced cell death and cognitive dysfunction (Xiao et al., 2021). Moreover, newer studies deduced that NF-α1 regulates BDNF function, as rectifying NF-α1 expression markedly increased the production of mature BDNF and restored neurogenesis in the aged brain (Jiang et al., 2024; Liu et al., 2023).

### 7.2 Nerve growth factor (NGF)

A number of phase 1 trials have been conducted for NGF gene therapy. The first study was conducted by Tuszynski et al. (2005) in which they implanted genetically modified autologous fibroblasts expressing NGF in the forebrain of patients with mild AD (Tuszynski et al., 2005). This study concluded that the NGF gene therapy promoted cholinergic neuronal sprouting, increased glucose metabolism, improved cognition and slowed down disease progression (Tuszynski et al., 2005). Further studies utilizing NGF gene therapy deduced improvement of cholinergic markers in CSF, cholinergic neuronal survival and cognition (Eriksdotter-Jönhagen et al., 2012; Eyjolfsdottir et al., 2016; Rafii et al., 2014). All studies reported the gene therapy to be safe and well-tolerated with no long-term adverse effects. Despite the positive results, the NGF gene therapy was stopped after phase 2 trial as it failed to show any cognitive improvement in its recipients (Castle et al., 2020). However, a recent study utilizing MRI-guided focused ultrasound (MRIgFUS) followed by the non-invasive delivery of NGF receptor agonist, TrkA agonist termed D3, to the brain has showed efficacy to improve ACh system by decreasing neurodegeneration of BFCNs and improving brain levels of ACh and Choline acetyltransferase. Besides, D3 delivery was associated with increased hippocampal neurogenesis and decreased amyloid plaque burden, thereby improving cognitive deficits in transgenic AD mice (Xhima et al., 2022). As the MRIgFUS helps to make the BBB permeable to the D3 delivery to brain parenchyma, this treatment outweighs other methods due to its non-invasive CNS delivery technique of AD modulation, and may form an effective therapy when combined with other nootropic drugs.

### 7.3 Neurotrophin-3

It was observed that neurotrophin-3 gene therapy improves the survival and differentiation of existing neurons and the growth and differentiation of new neurons (Yalvac et al., 2016). In a newer study, the transfection of bone marrow-derived mesenchymal stem cells (BMSC) with NT-3 was found to promote the differentiation of BMSC into neurons resulting in improved cognitive function in AD-induced rats (Yan et al., 2021). Similarly, injection of the adenovirus-associated viruses carrying NT-3 vector plasmids (AAV-NT3) into the hippocampus resulted in increased maturation of the hippocampal neurons. However, since this method suppressed the early neuronal processes, including neuronal proliferation, the AAV-NT3 therapy may negatively regulate the hippocampal neurogenesis in long-run, which can prove detrimental in the progression of neurodegenerative diseases like AD (Kasakura et al., 2023). Conversely, to our knowledge, no such gene therapy studies have been conducted targeting NT-4/5.

## 8 Discussion and future directions

### 8.1 Blood brain barrier and the multifactorial nature of AD

One of the foremost challenges in the therapeutic landscape for AD is the presence of the BBB that restricts the passage of neurotrophins into brain tissue. Conventional delivery methods, such as stereotactic injections, are invasive and carry inherent risks. Conversely, novel approaches like FUS show promise but remain largely experimental (Nagahara et al., 2018; Xhima et al., 2022), with the amount of therapeutic agent reaching the brain yet to be fully elucidated. Another obstacle in the development of gene therapies lies in the inconsistent outcomes observed in clinical trials. Although animal studies targeting neurotrophins have shown promising results, human trials have yielded mixed results. For instance, initial studies on NGF gene therapy indicated cognitive improvements in mild AD cases; however, subsequent trials failed to consistently replicate these findings (Castle et al., 2020; Eyjolfsdottir et al., 2016). Additionally, while short-term studies suggest neuroprotective benefits from gene therapy, long-term safety remains uncertain. Risks associated with immune responses, off-target effects, and the potential for ectopic expression (on systemic administration) of neurotrophic factors and long-term transgene expression of viral vectors could lead to toxicity and other unintended consequences (Sun and Roy, 2021).

Additionally, the multifactorial nature of AD further complicates therapeutic strategies. AD is a complex disorder encompassing intricate pathology, including the accumulation of amyloid plaques, the formation of tau tangles, synaptic dysfunction, and neuroinflammation. Thus, a single-target therapeutic approach may be insufficient. This complexity underscores the need for therapies that simultaneously target multiple pathways. Amid these concerns, the NF-α1 has emerged as a potential multi-target approach that can also modulate the function of other neurotrophins and dopaminergic system (Li et al., 2021; Park et al., 2008; Selvaraj et al., 2015; Zhang et al., 2009).

NF-α1 is known to enhance synaptic plasticity through the modulation of neurotrophic signaling pathways, thereby promoting neuronal survival and function (Xiao and Loh, 2022). Additionally, NF-α1 plays a significant role in mitigating neuroinflammation, a critical component of AD pathology, by regulating the activation of glial cells and the release of pro-inflammatory cytokines (Xiao et al., 2023; Jiang et al., 2024). Furthermore, NF-α1 has been implicated in supporting dopaminergic function, which is often compromised in AD and related neurodegenerative disorders (Zhang et al., 2009).

The ability of NF-α1 to concurrently influence these diverse pathways presents a unique advantage in addressing the complex pathophysiology of AD. To maximize therapeutic outcomes, it is advisable to consider combinatory approaches. For instance, pairing NF-α1 with amyloid-clearing antibodies, such as aducanumab or lecanemab, could potentially enhance amyloid-beta clearance while simultaneously promoting neuronal resilience through neurotrophic support (Van et al., 2023). Furthermore, the incorporation of neuroprotective agents like memantine or agents targeting mitochondrial dysfunction, such as mitochondrial stabilizers or antioxidants, could synergistically enhance neuronal survival and cognitive function (Ortiz et al., 2019).

Recent studies have shown that mitochondrial dysfunction contributes significantly to neurodegeneration in AD, and thus, therapies that support mitochondrial health may amplify the neuroprotective effects of NF-α1 (Arrozi et al., 2017). Additionally, tocotrienol-rich fractions (TRF) have been shown to modulate hippocampal gene expression in an AD mouse model, influencing pathways involved in oxidative stress, neuroinflammation, and neuronal survival (Nasri et al., 2019). The neuroprotective properties of TRF, including its ability to stabilize mitochondrial function and reduce neuroinflammation, suggest that combining it with NF-α1 could further enhance neuronal resilience by mitigating oxidative stress-induced damage, a key contributor to AD pathogenesis.

### 8.2 Translational challenges in NF-α1 gene therapy for AD

Being a newly identified neurotrophin effective against AD, its consistent result in rodents is still a question. Moreover, a similar efficacy in human brain is still unknown. Furthermore, its safety and adverse effects are yet to be evaluated. Rodent models remain indispensable in preclinical AD research. However, significant anatomical and functional differences between rodents and humans pose challenges to the translational success of therapies. Humans possess a more complex neocortex and larger brain size, which influence how neurotrophic factor-based therapies function. Additionally, the extensive synaptic connectivity and cortical organization in humans surpass those in rodents, potentially leading to variable therapeutic outcomes when scaling from animal studies to human clinical trials. Similarly, the immune system in rodents differs considerably from that of humans, particularly in terms of microglial activation and inflammatory responses. These discrepancies often result in rodent models inaccurately mimicking the chronic neuroinflammation characteristic of AD, limiting their reliability in predicting human responses to gene therapy (Gao et al., 2022).

The use of viral vectors, such as AAVs or lentiviruses to overexpress genes such as the secreted APP-alpha has been shown to be neuroprotective against amyloid or tau pathology (Tan et al., 2018). Its augmentation with NF-α1 gene could augment its neuroprotective and nootropic effects. This however, introduces notable safety concerns. One major risk is insertional mutagenesis, where the integration of the viral vector into the host genome disrupts critical genes or regulatory regions. Such unintended integration can lead to harmful outcomes, particularly concerning in chronic conditions like AD that may require long-term or repeated therapy (Piguet et al., 2017). Another challenge is ectopic expression, where the transgene is expressed in unintended tissues or at inappropriate levels. This aberrant expression can result in toxicity and dysregulation of cellular homeostasis, potentially exacerbating disease progression. Additionally, immune reactions remain a critical barrier, as viral vectors can provoke inflammatory responses that damage tissues or reduce the efficacy of gene delivery. Patients with pre-existing immunity to the viral vector face diminished therapeutic outcomes, often requiring immunosuppressive therapies that carry additional risks (Butt et al., 2022).

### 8.3 Mitigating strategies

Efforts to mitigate these risks have centered on the development of safer and more precise delivery systems. Non-viral platforms, such as lipid nanoparticles or electroporation-based delivery systems, are gaining traction as alternatives to viral vectors (Hou et al., 2021; Kitte et al., 2023). These methods enable transient gene expression, reducing the likelihood of insertional mutagenesis and offering safer options for repeated dosing. Another approach involves transient expression systems, such as mRNA- or plasmid-based therapies, which provide temporary therapeutic effects without the long-term risks associated with viral vector integration (Pagant and Liberatore, 2021). Lastly, immune-modulating therapies aim to mitigate adverse immune responses during treatment. For instance, immunosuppressants or engineered antibodies targeting activated immune cells may help control inflammation while preserving the therapeutic benefits of NF-α1 gene therapy (Pardi et al., 2018).

In summary, while significant challenges remain in the realm of AD therapeutics, ongoing research is paving the way for innovative treatments that may ultimately improve patient outcomes and quality of life. That being said, NF-α1 therapy can prove to be an effective treatment having the potential to halt the disease progression and improve cognitive impairment. Continued optimization of delivery systems, evaluation of the preferred route of administration, and improved comprehension of the mechanism of action will be vital for enhancing targeted treatment and ensuring sustained gene expression.
